# Successful replantation of an amputated penis: a case report and review of the literature

**DOI:** 10.1186/1752-1947-8-125

**Published:** 2014-04-09

**Authors:** Omar Riyach, Aziz El Majdoub, Mohammed Fadl Tazi, Jalal Eddine El Ammari, Mohammed Jamal El Fassi, Abdelhak Khallouk, Moulay Hassan Farih

**Affiliations:** 1 Department of Urology, University Hospital Center Hassan II-FES, Fes, Morocco; 2 Faculté de Médecineet de Pharmacie de Fès, BP: 1893, Km 2.200 Route de Sidi Harazem Fes, Fes, Morocco

**Keywords:** Amputation, Penis, Reimplantation

## Abstract

**Introduction:**

Amputation of the penis is a rare traumatic injury reported from various parts of the world as isolated cases. A complete reconstruction of all penile structures should be attempted in one stage which provides the best chance for full rehabilitation of the patient.

**Case presentation:**

We report the case of a 35-year-old Berber man who was admitted at the Emergency Department for incomplete criminal amputation of his penis, which was successfully reattached by using a macrosurgical technique. After surgery, near-normal appearance and function including a good urine flow and absence of urethral stricture, capability of erection and near normal sensitivity, were observed.

**Conclusions:**

The importance of using macrosurgical reimplantation in incomplete penile amputation in order to achieve better functional and cosmetic results is discussed. In addition, we also highlight the potential anatomical role of corpus spongiosum in the arterial and venous blood supply to the penis.

## Introduction

Penile amputation is a rare situation in daily urological practice. In the majority of cases it occurs on psychotic grounds but it may be secondary to the abuse of drugs or alcohol or it can be caused by other people’s actions such as violence and crime [1,2]. Treatment and care vary depending on the severity of the lesions, the consultation delay and the patient's mental state. We report a case of criminal penile amputation. Through this observation, and a recent literature review, the authors analyze the aspects, management and outcome of this urological injury.

## Case presentation

**Figure 1:**
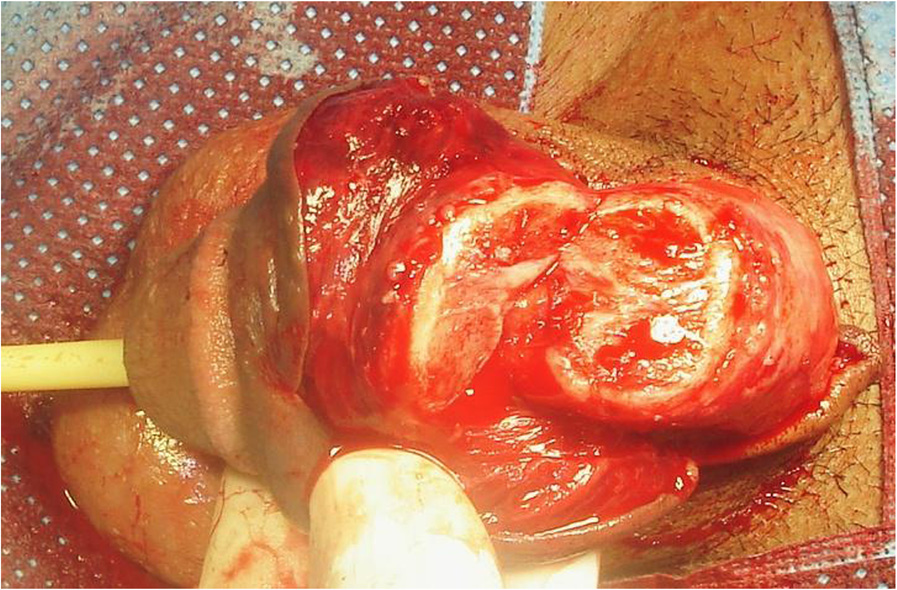
Incomplete amputation of penis.

**Figure 2:**
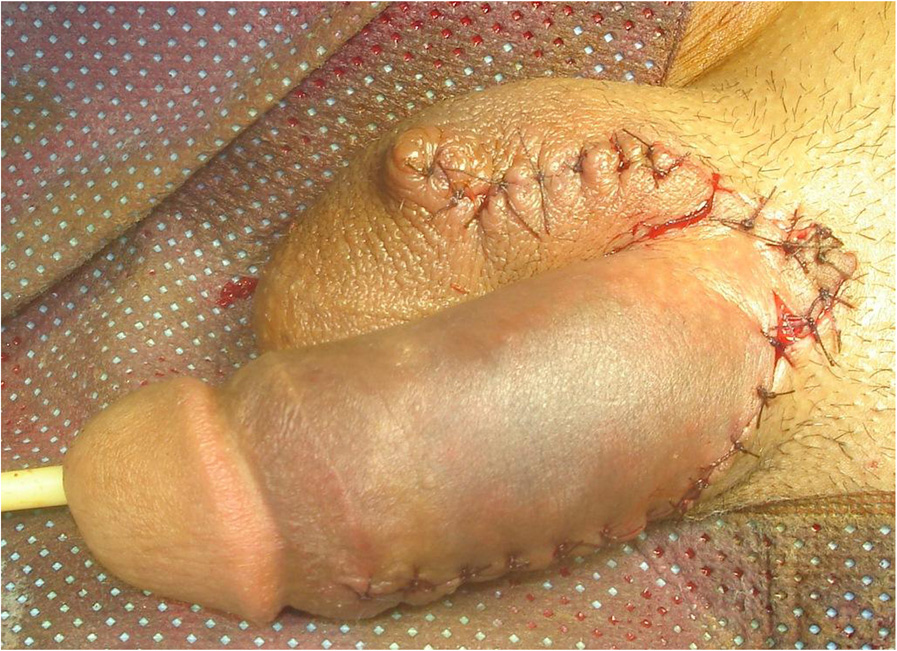
The replantated penis showed considerable edema of the skin, of the penile shaft and the prepuce.

**Figure 3:**
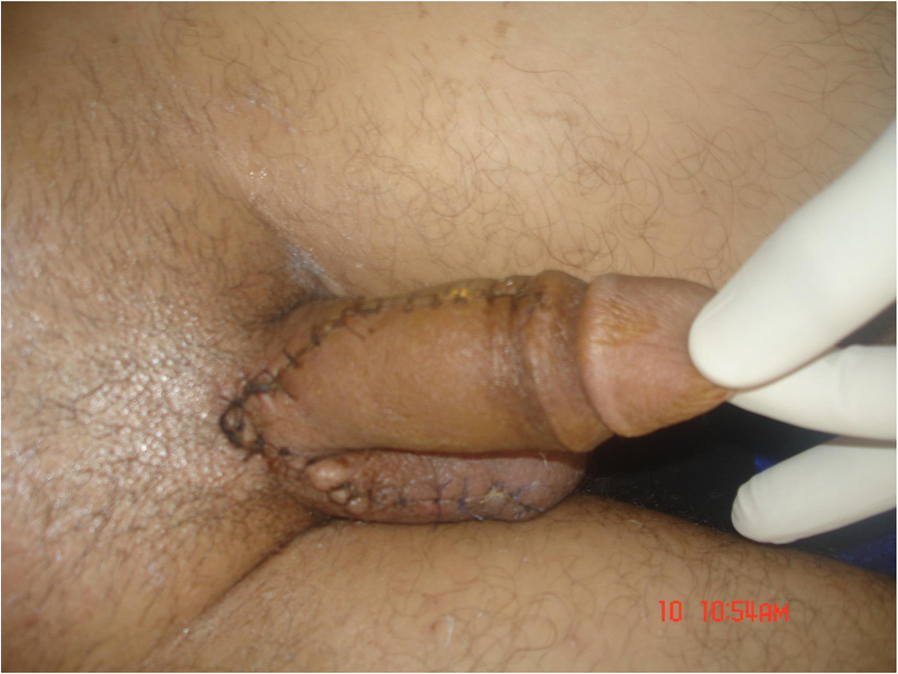
A normal-appearing penis.

In December 2012, a 35-year-old Berber man, without any past medical history, presented to our Emergency Department with incomplete amputation of his penis after being assaulted by an unknown actor who cut off his penis using a shaving blade 3cm distal from the mons pubis. A physical examination did not show any other remarkable finding. There was a clear cut through his cavernosal bodies with diffuse bleeding from the dorsal vessels (Figure 1). His scrotum and testicles were found to be intact. After thorough ablution with Ringer’s lactate solution and an antitetanic injection he was admitted to the Operation Room. As he had lost blood before his admission, he was transfused with two units of red blood cells during reimplantation. He was placed under general anesthesia. A rubber band was placed, as a tourniquet, around the proximal end under his pubis for bleeding control. A 16Fr. silicone catheter was inserted transurethrally through the distal amputated part followed by the anastomosis of his urethra and the cavernosal bodies. His urethra was repaired by end-to-end anastomosis using interrupted 4/0 synthetic absorbable sutures. The tunica albuginea of corporal bodies was repaired circumferentially with 3/0 vicryl. His superficial deep dorsal veins as well as his deep penile arteries were not repaired. As a last step his Buck's fascia was closed with 3/0 vicryl and the skin with 3/0 nylon (Figure 2). Total ischemia time was about 6 hours. The Foley catheter was removed after 4 weeks postoperatively with good urine flow. On follow-up examination, 5 weeks later, no necrosis was noticed on his skin; there was a normal-appearing penis (Figure 3) without difficulty in voiding and good sensation. He reported the restoration of his penile erection and ejaculation during sexual intercourse.

## Discussion

The majority of penile amputations are caused by self-mutilation. A minority of the reported cases are masturbatory trauma, accidental or industrial trauma, and attacks by spouses in retaliation for unfaithfulness. In our case it was a criminal amputation using a shaving blade. The first documented case of macroscopic penile replantation was reported in 1929 by Ehrich [3]. A review of the literature revealed at least 30 cases of penile autoamputation with successful replantation [3,4] since 1970.

Many factors contribute to favorable final outcomes: the degree of injury, type of injury (crushed, lacerated, or incised), duration of warm ischemia, the equipment used, and experience of the operative team [5]. Analysis of our case revealed that the cleanly incised injury, with incomplete section of penis involving both corpora cavernosa and the spongy body, with a short duration of cold ischemia were the important factors that influenced the outcome.

A maximum of 6 hours was conventionally accepted to attempt reimplantation, while the use of microsurgery gave the opportunity for successful operations after 16 or even 24 hours of ischemia [6,7].

The macrosurgical replantation of the penis depends on corporal sinusoidal blood flow with the distal amputated part, as a composite graft leads to high complication rates of skin necrosis, fistula formation, loss of sensations and erectile dysfunction. In contrast, the microsurgical technique of anastomosing the penile shaft structures provides early restoration of blood flow with the best prospects for graft survival, normal erectile function and optimal benefits with fewer complications [8-10]. Approximately 40 cases of penile reattachments using nonmicrosurgical techniques have been published [7]. In our case study we demonstrated that even without venous drainage restoration, good postoperative results can be obtained if a part of the corpus spongiosum is spared. Frequent complications included necrosis of the distal glans and skin, stricture, and fistula. No complications were reported in our case. Most authors recommend urinary diversion by suprapubic cystostomy, but we did not find this necessary in our patient.

This case raises a curiosity about the probable anatomical role of corpus spongiosum in the arterial supply and venous drainage of the penis as well as the erection.

## Conclusions

Penile amputations are extremely rare. This case outlines an approach to the assessment and treatment of penile amputation with partial spongiosum injury. We demonstrated that a macrosurgical technique without venous drainage restoration is able to restore normal erectile and urinary function with acceptable outcome in incomplete penile amputation with partial corpus spongiosum injury.

## Consent

Written informed consent was obtained from the patient for publication of this manuscript and accompanying images. A copy of the written consent is available for review by the Editor-in-Chief of this journal.

## Competing interests

The authors declare that they have no competing interests.

## Authors’ contributions

OR, the principal author, made major contributions in writing the manuscript. AE, MFT, JE, MJE, AK and MHF analyzed and interpreted the patient data and the reviews of the literature. All authors read and approved the final manuscript.
